# Increasing access into higher education: Insights from the 2011 African Network on Evidence-to-Action on Disability Symposium – Education Commission

**DOI:** 10.4102/ajod.v3i2.78

**Published:** 2014-06-04

**Authors:** Marcia Lyner-Cleophas, Estelle Swart, Tsitsi Chataika, Diane Bell

**Affiliations:** 1 Centre for Student Counselling and Development, Stellenbosch University, South Africa; 2 Department of Educational Psychology, Stellenbosch University, South Africa; 3 Department of Educational Foundations, University of Zimbabwe, Zimbabwe; 4 Academic Affairs, Stellenbosch University Business School, South Africa

## Abstract

This article provides some insights into the challenges regarding inclusion in higher education of students with disabilities. It does this by elucidating aspects of the proceedings of the Education Commission at the African Network on Evidence-to-Action on Disability (AfriNEAD) Symposium, which took place in Zimbabwe in November 2011. The presentations specifically focused on the education of people with disabilities from early childhood through to higher education. This article, however, is informed by presentations focusing on increasing access to higher education. The article is focused on the implementation of evidence in practice, research and policies stemming from rigorous debate and scientific foundations, whilst taking into account the dynamic realities of the higher education context. Themes such as the systemic approach needed for inclusion to be successful, increasing access and the dynamic role of students with disabilities are highlighted.

## Introduction

Access to higher education for people with disabilities presents opportunities as well as challenges. Since the move towards inclusion after the introduction of the *Salamanca statement and framework for action on special needs education* (United Nations Educational, Scientific and Cultural Organization [UNESCO] 1994), higher education has had to grapple with a range of new developments on national and local levels to meet the increasing influx of students continuing into the tertiary education sector. Higher education endeavours to promote inclusion and the participation of a diverse student group, but faces several challenges. The onset of the global human rights discourse during the latter part of the 20th century has brought about new ways of having to address marginalised communities, such as people with disabilities. It has become necessary to review current educational practices; hence the call for inclusivity and participation, which are vital for human dignity and human rights. The South African *White paper on post-school education and training* (Department of Higher Education and Training 2013) notes that despite national attempts at policies that include people with disabilities, higher education still manages disability support in a fragmented way, as if it is separate from existing transformation and diversity programmes. This policy notes the importance of including support staff, management and lecturers in the process of disability inclusion, thus pointing to a systemic approach to inclusion.

Research shows that education is a major determinant of an individual’s societal status and social mobility, as it influences career prospects, and that higher education is considered a gateway to a better future (Chataika 2010). However, for social mobility to be realised, society and its range of systems need to be galvanised to think and act inclusively.

The presentations discussed in this article highlight four themes: the promotion of inclusive practices; the importance of a systemic approach to facilitate inclusive practices; ways of increasing access to higher education; and the dynamic role of the individual in interaction with the systems. The authors share ways of increasing access for students with disabilities in higher education by highlighting the systems involved in the process. They also point out that the facilitation of such access involves a range of role players in the various systems, including the changed role of educational psychologists. The authors also emphasise the resilience of individuals in the process of inclusion, but caution against students having to battle against the many systems in higher education to achieve inclusion.

The kind of integration of support that fosters inclusion with a focus on the range of systems is conceptualised well within Bronfenbrenner’s bio-ecological model (Ceci 2006). Bronfenbrenner highlights four dimensions when contextualising a person; according to him, people’s experiences are influenced by the context of time, space, processes and personal characteristics. These have a dynamic effect on each other and take place on various levels in society. The micro-level involves the person, whilst the meso-level involves other systems such as education. At the macro-level, policies influence what happens on the other levels. The four themes noted in the previous paragraph are well placed within this model as these are impacted by the range of ecological systems within which people function.

## Increasing access of students with disabilities in higher education

Traditionally, limited attention has been placed on addressing issues of access, retention, progression and participation of students with disabilities within higher education institutions (HEIs). The Disability in Higher Education Project, conducted by the Foundation of Tertiary Institutions of the Northern Metropolis (FOTIM) from 2009 until 2011, therefore focused on describing and analysing the role and functioning of specialised disability units at various universities in South Africa and how they interact with the various systems within the universities to foster inclusive practices (Bell 2011).

The FOTIM research project included data from 15 disability units at 23 HEIs in South Africa. A key finding of this project was that disability units in most universities formed part of student counselling services or the Student Affairs Department, which made their autonomous functioning difficult. This influenced their effectiveness as they were not given sufficient independence to develop relevant programmes. The association with student counselling services often reinforces the medical model of disability which seeks to pathologise, rehabilitate and remediate people with disabilities without focusing on disabling environments. More autonomous functioning could foster better campus-wide communication and more interaction with other departments and systems that students interact with, given the cross-cutting nature of disability. Other important insights included the continued inaccessibility of key buildings such as the library, the lack of awareness of university staff regarding disability policies and practices, and inadequate funding from the Department of Higher Education and Training to promote access and inclusion (FOTIM 2011:45). These are some of the critical systems within which successful inclusion can take place.

One of the project’s recommendations was that functional independent disability units were essential in order to facilitate the inclusion of students with disabilities since faculties and departments struggle to deal with this in isolation. Autonomous disability units, with a direct reporting line to the registrar or deputy vice-chancellor, would allow for the participation of academics at a higher level of negotiation for necessary resources and awareness, which could help to effect meaningful inclusion of students in the various faculties. The Department of Higher Education and Training should explore appropriate funding mechanisms to financially assist universities to enable the promotion of disability inclusion through accommodating and including students (and staff) with disabilities. Although there is a strong legislative and policy framework in South Africa, implementation is slow. A further recommendation based on international experience speaks to the need for a specific disability anti-discriminatory act, which should be enacted at a national level in order to raise the profile of disability issues and bring them to the fore as a compliance imperative (FOTIM 2011:21).

The FOTIM research report concludes that it is critical that the disability agenda needs to be entrenched in the way in which HEIs function as a whole. Also, in order to promote access to higher education, disability inclusion must be fully embedded in the overall functioning of the university at all levels.

## Realigning the educational psychologist’s role to promote access into higher education

Lyner-Cleophas and Swart (2011) examined the role of the educational psychologist as a role-player in the promotion of human rights and inclusion, guided by the revised practice framework of the Health Professions Council of South Africa (HPCSA) (Department of Health 2011). They argue that educational psychologists can play an important role in developing inclusive practices in education.

Traditionally, the role of educational psychologists was mainly to diagnose learning problems, facilitate educational placements and to assist with ‘remedies’ so that learners would fit into the system. These roles were embedded in the medical model of disability referred to earlier, which is deficit-based and exclusionary (Oliver 2009; Swartz & Watermeyer 2006). Educational psychologists are currently realigning their role to work within a bio-ecological model where the focus is within the range of systems and the dynamic interaction between these systems and its impact on persons with disabilities. These professionals work with learners across their life-span, and can therefore support the transitioning of learners with disabilities from secondary school to higher education from a bio-ecological perspective. Their role within Bronfenbrenner’s bio-ecological framework (Ceci 2006) involves preparing the learner as well as the various systems in higher education, at a micro-, meso- and macro-level.

Educational psychologists are strategic role-players in developing support and inclusive practices in higher education, together with campus departments (academic, support and administrative staff). They can also influence strategies and policy at a national level by way of bodies like the Higher Education Disability Services Association (HEDSA 2010) and the HPCSA. These play a strong advisory and developmental role in the transformative and inclusive way of working, which aims at re-conceptualising values and beliefs that celebrate diversity. Advocacy and community education have become very important as well as the development role when transitioning into the various educational phases, including higher education. Educational psychologists can also play a vital role in the training of staff regarding inclusion in higher education.

## Struggles and coping mechanisms of students with disabilities in higher education

Part of the bio-ecological framework framing our thinking is the position of the individual at a micro-level, which should not be underestimated. Chataika (2010) studied personal experiences of students with disabilities in higher education in Zimbabwe. She established that they continue to face attitudinal, physical and institutional barriers. The study further revealed that the students have the ability to develop coping mechanisms that help them reach their educational goals. A positive attitude and self-advocacy skills were seen as the most important factors in determining the success of students with disabilities in higher education. Similarly, self-determination or self-belief was seen as a vehicle to success. However, Chataika (2011) calls for improved policy and practice to ensure meaningful disability inclusion in education, without students with disabilities becoming ‘superheroes’ who spend most of their time trying to surmount a myriad of barriers that are common in most universities.

## Conclusion

In this article, we briefly presented some of the insights gained from the Education Commission at the 2011 AfriNEAD Symposium. Evidence of challenges in implementing inclusive practices systemically in higher education, ranging from a policy environment without monitoring and oversight functions, the under-involvement of disability units, to challenges with physical infrastructure and funding, were shared. Recommendations for improving inclusive practices and access to higher education for students with disabilities included improved policy development and practices across bio-ecological systems such as government, university support departments and faculties. There is also a need to monitor policy implementation across university campuses in Africa. The various systems need to work together to promote an inclusive higher education experience for students that is accessible on a range of levels.

The process of inclusion takes time, and the resilience of students who engage in self-advocacy in the process cannot be underestimated. However, they may not serve as a reason for stalling inclusivity across campuses. Inclusion can be realised effectively when policy-makers and universities develop related policies by including relevant stakeholders to ensure campus-wide inclusive practices.

Our hope is to see policy-makers, people with disabilities, academics, researchers and other stakeholders earnestly engage with issues raised in this article. This will subsequently contribute to the theoretical concept, increased access and meaningful inclusion and participation of students with disabilities in higher education through the development and enactment of inclusive legislation in Africa. Ultimately, we hope that increased enrolment of students with disabilities in higher education will be realised.
